# Evaluating Text-to-Image Generated Photorealistic Images of Human Anatomy

**DOI:** 10.7759/cureus.74193

**Published:** 2024-11-21

**Authors:** Paula Muhr, Yating Pan, Charlotte Tumescheit, Ann-Kathrin Kübler, Hatice Kübra Parmaksiz, Cheng Chen, Pablo Sebastián Bolaños Orozco, Soeren S Lienkamp, Janna Hastings

**Affiliations:** 1 Faculty of Medicine, Institute for Implementation Science in Health Care, University of Zurich, Zurich, CHE; 2 Digital Society Initiative, University of Zurich, Zurich, CHE; 3 Faculty of Medicine, Institute for Anatomy, University of Zurich, Zurich, CHE

**Keywords:** ai in medicine, anatomical education, diffusion model, generative ai, large multi-modal model, medical image, photorealistic synthetic anatomical image, text-to-image generation

## Abstract

Background: Generative artificial intelligence (AI) models that can produce photorealistic images from text descriptions have many applications in medicine, including medical education and the generation of synthetic data. However, it can be challenging to evaluate their heterogeneous outputs and to compare between different models. There is a need for a systematic approach enabling image and model comparisons.

Method: To address this gap, we developed an error classification system for annotating errors in AI-generated photorealistic images of humans and applied our method to a corpus of 240 images generated with three different models (DALL-E 3, Stable Diffusion XL, and Stable Cascade) using 10 prompts with eight images per prompt.

Results: The error classification system identifies five different error types with three different severities across five anatomical regions and specifies an associated quantitative scoring method based on aggregated proportions of errors per expected count of anatomical components for the generated image. We assessed inter-rater agreement by double-annotating 25% of the images and calculating Krippendorf’s alpha and compared results across the three models and 10 prompts quantitatively using a cumulative score per image. The error classification system, accompanying training manual, generated image collection, annotations, and all associated scripts, is available from our GitHub repository at https://github.com/hastingslab-org/ai-human-images. Inter-rater agreement was relatively poor, reflecting the subjectivity of the error classification task. Model comparisons revealed that DALL-E 3 performed consistently better than Stable Diffusion; however, the latter generated images reflecting more diversity in personal attributes. Images with groups of people were more challenging for all the models than individuals or pairs; some prompts were challenging for all models.

Conclusion: Our method enables systematic comparison of AI-generated photorealistic images of humans; our results can serve to catalyse improvements in these models for medical applications.

## Introduction

Generative artificial intelligence (AI) is poised to transform medicine through a range of potential future applications [1,2,3]. However, the evaluation of AI-generated outputs poses significant challenges [4], as generated content is heterogeneous and subject to biases [5,6], and it is difficult to compare different models when the outputs are heterogeneous with no underlying ground truth. While the bulk of research into the impact of generative models in medicine has thus far focused on the generation of natural language texts, multi-modal models such as those that generate images promise an even greater impact [3]. The focus of the current study is on text-to-image generative models, such as DALL-E and Stable Diffusion, which enable the creation of photorealistic images from natural language descriptions. These models are trained on large datasets of combinations of text descriptions and accompanying images, and thereby they gain the capability to synthesize a corresponding image for a given text description. Applications of these models in medicine include generating representative illustrations for medical education, particularly in anatomy [7], and creating synthetic data for a variety of scientific purposes in contexts where real-world images are insufficient, lacking, or unavailable due to privacy concerns. While task-specific models for image generation, e.g., using generative adversarial networks (GANs) [8,9], have been available for some time, the newer diffusion-based text-to-image models are distinguished by their generality and ease of use and have shown better performance on a range of image types [10].

The potential of these text-to-image generative models in medicine is already being evaluated; for example, they have been evaluated for their applicability to generating accurate anatomical illustrations of the human skull, heart, and brain [7], human faces, and other body parts with various pathologies [11,12,13], and illustrations of resuscitation techniques [14,15]. Their applicability has also been tested for illustration, surgical planning, and patient consultation in surgery, dental surgery [16], and aesthetic surgery [17]. Moreover, DALL-E 2 has been used to synthesize realistic images for training predictive algorithms in dermatology [18] and for aphasia assessment [19]. The technology has also been implemented to generate synthetic radiology images, e.g., chest X-rays [20,21].

It has been observed that a variety of visual errors are common in text-to-image generated images, in particular, when rendering human hands [22]. Such inadvertent errors may potentially blur the diagnostic boundaries between the anatomical representations of healthy and diseased anatomy, which are essential for downstream applications in medicine. However, thus far, there has been no systematic examination of such errors or their direct comparison across different generative models. Evaluation of such images follows a range of different standard metrics for image quality and for alignment between text and image [23], as well as aspects such as toxicity and fairness [24], but these metrics do not specifically address anatomical or medical correctness. To address this gap, our study introduces a novel method of descriptive evaluation of anatomical errors in generated photorealistic images of the human body. Importantly, our focus on assessing model-inherent errors in generative photorealistic imagery of human bodies has no intention of discriminating against people with non-normative bodies. Our assumption is that understanding the errors that these models make on the kinds of everyday images they were trained on will be essential for future evaluation of the errors these models make when generating domain-specific medical images. Our study generates a framework for evaluating and comparing the errors in generated images that are transferable to other anatomical images, by identifying general categories of errors such as missing parts or abnormal configurations, and by introducing a scoring system that can be used for comparisons. In the current study, we apply our method to generated images of humans in the present study, but in future works, we plan to apply the method to other anatomical imaging modalities, including retinal fundus images, for which synthetic images are valuable to supplement the training data for diagnostic predictive models. In addition, our method enables comparisons between models and between model versions, which has the potential to drive forward innovations in medical generative AI. 

This article was previously posted as a preprint to the medRxiv preprint server on August 21, 2024 (https://doi.org/10.1101/2024.08.21.24312353).

## Materials and methods

Development of the anatomical error classification system

We developed the anatomical error classification system iteratively, as extensively detailed in Appendix 1. In brief, we generated and annotated a collection of photorealistic images sampled from three models and multiple prompts and then applied our initial classification system to compute inter-rater reliability, as well as to compare models and prompts, followed by improving the system iteratively. The first iteration of the classification system defined five types of errors (missing, extra, configuration, orientation, and proportion) and five anatomical regions (torso, limbs, feet, hands, and face). In the second iteration, we introduced a proportion-based quantification of the errors in each image (Equation 1). In the final iteration, our quantitative system was extended with a weighting of errors by severity across three error severity levels ("a": low, "b": medium, and "c": severe) along with a cumulative error severity score per image (Equation 2).

Equation 1 (Proportion of Errors)

For each picture, each body part, each error type, and each error severity, we define the proportion of errors as the number of body parts *i *that present with the error type *j *with the corresponding error severity $k(n_{ij})_{k})$ divided by the number of body parts present, or the number of body parts that should be present given the number of people and what is or should be visible in the picture $(m_{ij})$: 

$PE_{(ij)_{k})} = \frac{n_{(ij)_{k})}}{m_{ij}}$ 

Equation 2 (Cumulative Score) 

Let $PE_{(ij)_{k}}$ be the number of body parts *i* that exhibit an error of type *j* of error severity *k*. Let $w_{a}$,$w_{b}$,$w_{c}$ be the error severity weights that correspond to the error severity a, b, and c, respectively (we used 0.2 for "a," 0.5 for "b," and 1 for "c," to down-weight less severe errors). Then, the cumulative score $C_{x}$ of image x is defined as

$C_{x} = \sum_{i=1}^{5} \sum_{j=1}^{5} (w_a . PE_{(ij)_{a}} + w_b . PE_{(ij)_{b}} + w_c . PE_{(ij)_{c}})$ 

Generating the image dataset

We used three models: DALL-E 3 (a commercial model, accessed via the ChatGPT online web interface, https://chat.openai.com/) and Stable Diffusion XL and Stable Cascade (two models from the non-commercial Stable Diffusion family, accessed via the web interfaces made available at HuggingFace, https://huggingface.co/spaces/google/sdxl and https://huggingface.co/spaces/multimodalart/stable-cascade, respectively).

We designed 10 prompts, covering a variety of visual scenarios presenting either individuals, couples, or groups of about five people in dynamic action and mutual interaction across heterogeneous everyday settings. The 10 prompts were as follows: 1) athlete performing salto, 2) person jogging, 3) mother (or father) holding a baby, 4) couple hugging, 5) two men (or women) wrestling in an arena, 6) old couple in a sauna, 7) physician examining a patient, 8) people eating pizza, 9) five people sunbathing on a beach, and 10) five people playing volleyball.

For each prompt, we generated eight images. We focused on obtaining a gender-balanced dataset: when a generative model showed implicit bias toward a particular gender, we explicitly prompted to counter this bias. We also focused on obtaining a dataset that covered a range of age groups, from babies to older individuals, and included diverse ethnicities, although most images nevertheless represent Caucasian individuals. 

Allocating image annotations

Our annotation team consisted of four annotators. To assess the inter-rater agreement between the annotators, we submitted 25% of our dataset (60 images) to double annotation. This resulted in 300 annotations that were equally distributed across the annotators. To ensure that the three generative models, the 10 prompts, and the 60 double-annotated images were randomly distributed across the four annotators, we automated the distribution (the script is available from our GitHub repository at https://github.com/hastingslab-org/ai-human-images). 

Assessing inter-rater reliability

We assessed the inter-rater agreement by calculating Krippendorff’s alpha coefficient [25] using the Python package fast-krippendorff. We applied Krippendorff’s alpha to three variations of our annotation results: 1) the cumulative error score for each image; 2) an overall error severity for each image (low, medium, high); and 3) a vector of 25 entries for each category and body part with 0 for no error and 1 for an error. We determined an overall error severity by using the cumulative score to categorize each image based on quantiles of the error distribution of each individual annotator: with the 0.5 quantile as a threshold for the overall error level *low*, 0.75 for overall error level *medium*, above 0.75 as overall error level *high*.

Quantitative model comparison

We applied the error classification system to several different comparisons using our image dataset: between models, between error types, between anatomical regions, and between individual vs. group prompts. For these comparisons, we used the cumulative score and the aggregate counts per error severity to compare the models.

## Results

The anatomical error classification system

**Figure 1:**
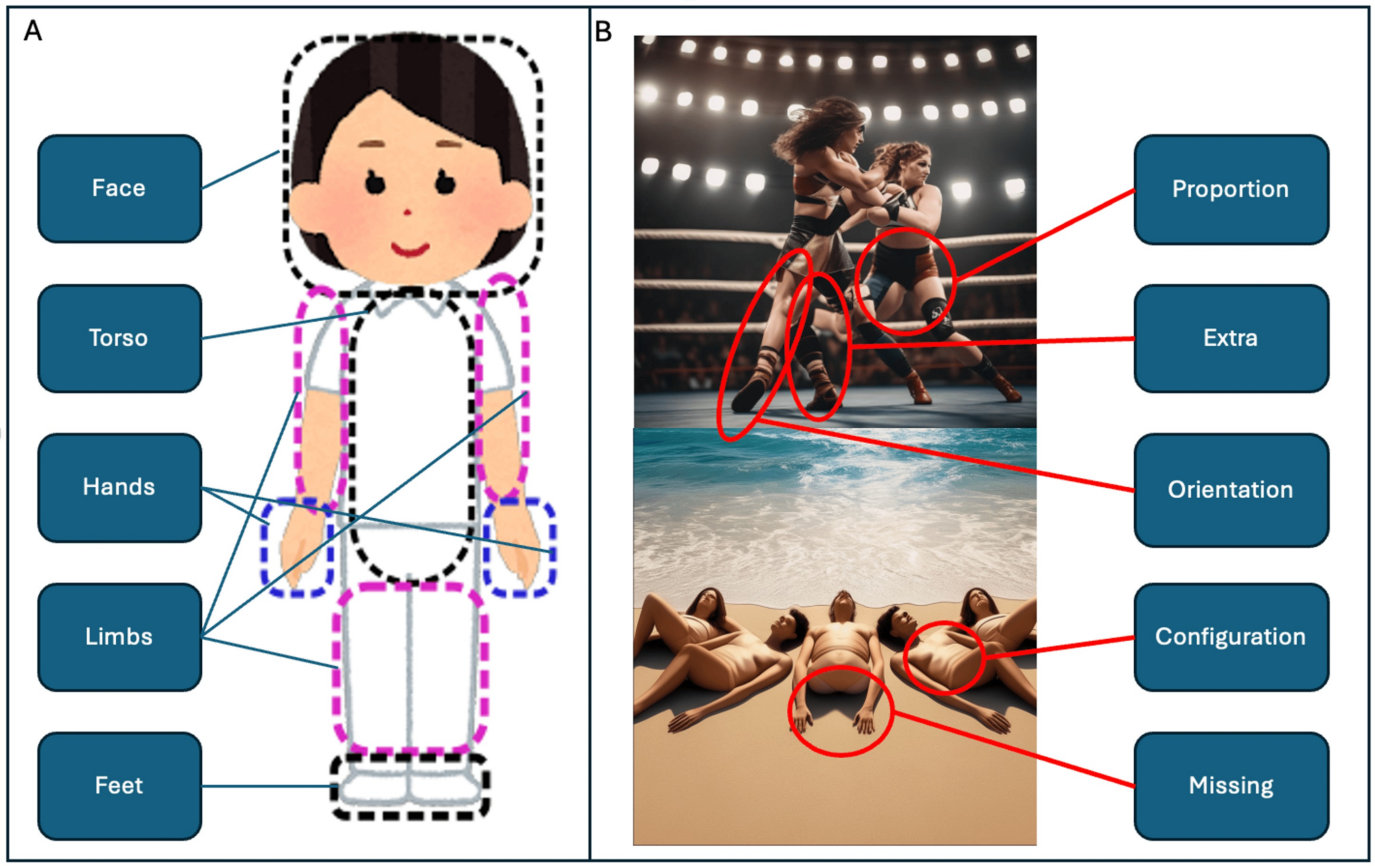
The anatomical error classification system Illustration of visual components (A) – anatomical regions; (B) – types of error. Figure is original work created for the present study; illustrated generated images are selected from the study-associated generated image dataset.

The anatomical error classification system (Figure 1) supports the identification and quantification of different anatomical errors in photorealistic images of humans based on a visual analysis of individual images.

**Figure 2:**
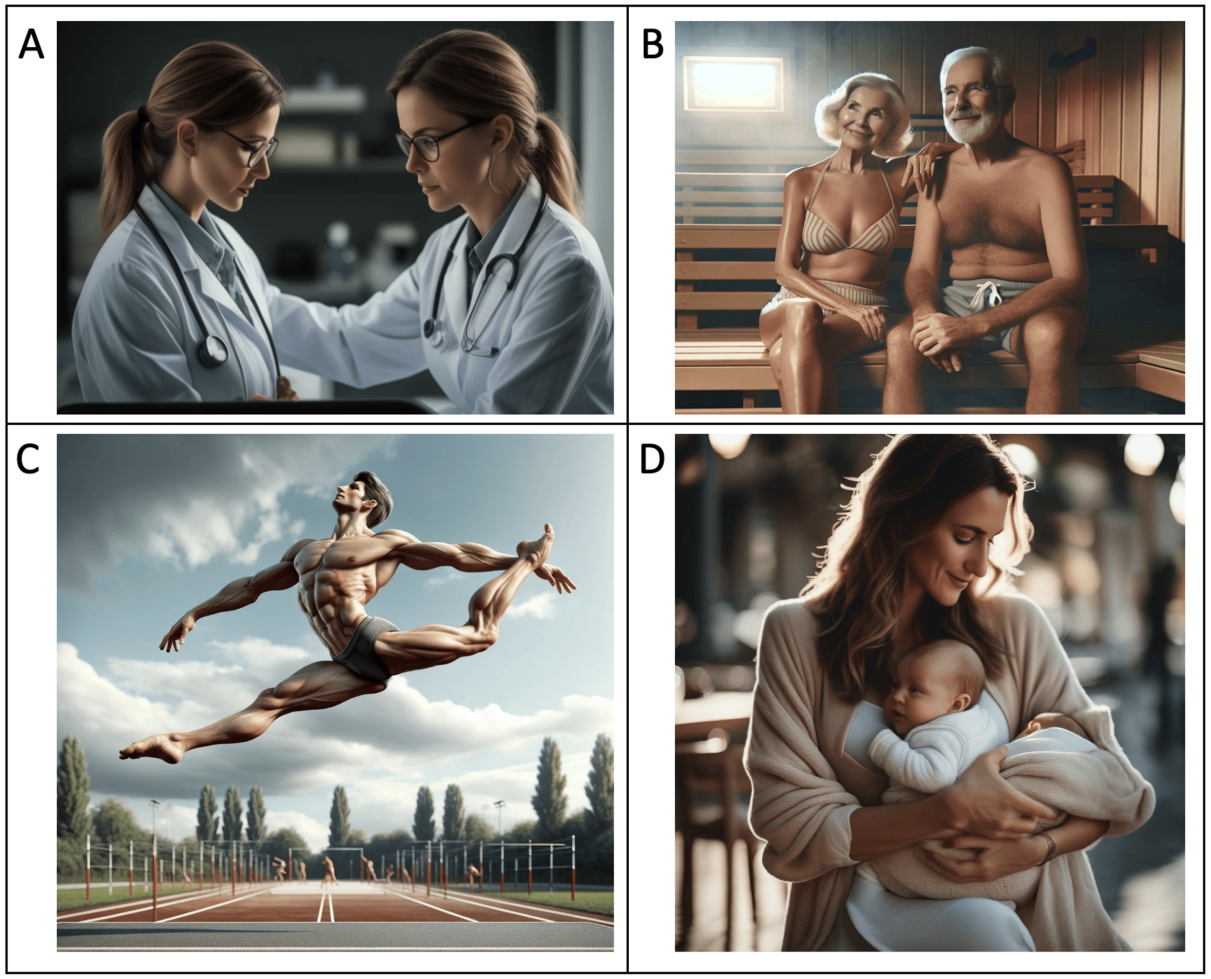
Selected example images from the generated image dataset Images selected from the study-associated generated dataset corresponding to the following prompts: (A) "physician examining patient"; (B) "old couple in sauna"; (C) "athlete performing salto"; (D) "mother holding baby". Note that the generated image may deviate from the provided prompts in some cases, e.g. A shows two physicians rather than a physician and a patient. This figure is an original work created for the present study.

The system consists of three components: (1) five anatomical regions, i.e., face, torso, hands, limbs, and feet (Figure 1A); (2) five error types, i.e., proportion, extra, orientation, configuration, and missing (Figure 1B); and (3) a quantification of error severity and a method of aggregation. The five error types are as follows: 1) *proportion errors*:* *misshaped or disproportionate body parts (e.g., limbs that are too short for the body); 2) *extra errors*: additional body parts (e.g., a hand with six fingers); 3)* orientation errors*:* *anatomically implausible orientations of body parts (e.g., the upper and lower body facing in opposite directions); 4*) configuration errors:* body parts that are disjointed (e.g. a hand disconnected from the body), displaced (e.g., a hand connected to the chest), or fused in ways that make their differentiation challenging (e.g., a hand merged with a fork); 5*) missing errors: *absent body parts (e.g., an arm without a hand).

We developed an annotation manual (available from our GitHub repository at https://github.com/hastingslab-org/ai-human-images) to mitigate inconsistent decisions among annotators. The manual introduces and explains the system using annotated examples. It instructs the annotators to only consider the main anatomical errors in generated images evident at first glance while disregarding minor errors that require zooming in to detect.

Image dataset and qualitative evaluation

Our three models, 10 prompts, and eight images per prompt resulted in a dataset of 240 generated images, also available from our repository. Example images are shown in Figure 2. 

While developing our image dataset, we noticed that some of the designed prompts proved to be challenging for some of the models. For example, the prompt about an aged couple in a sauna repeatedly led to an error in DALL-E 3 and even to the temporary blocking of the account for a few hours without any explanation. We had a similar experience with the prompt that instructed DALL-E 3 to generate two women wrestling in an arena, but not when prompting for wrestling men. The other two models did not have any problems with these prompts. Initially, we planned to use the prompt "a couple kissing." However, DALL-E 3 declared this forbidden content, so we adjusted the prompt to "a couple hugging."

We also noticed that when prompting the models to generate images that included two individuals, these individuals often looked almost identical, like twin or digital copies of each other. Another pattern that became apparent was that doctors always had a stethoscope, a detail that was impossible to avoid even when using negative prompting. Additional stereotypes included mothers being predominantly young and always having long hair, whereas older people, despite having somewhat wrinkled faces, were generated with bodies having impeccably smooth skin. Relatedly, across all models, the prompt ‘a couple hugging’ resulted in images of heteronormative couples.

Ethnic diversity was considerably more present in Stable Diffusion XL and Stable Cascade than DALL-E 3. When prompted to generate an image of a person without any explicit specification of gender, Stable Diffusion XL and Stable Cascade generated more gender-diverse images, whereas we had to prompt DALL-E 3 explicitly about gender aspects to generate a gender-balanced dataset.

Annotation results and quantitative evaluation

**Figure 3:**
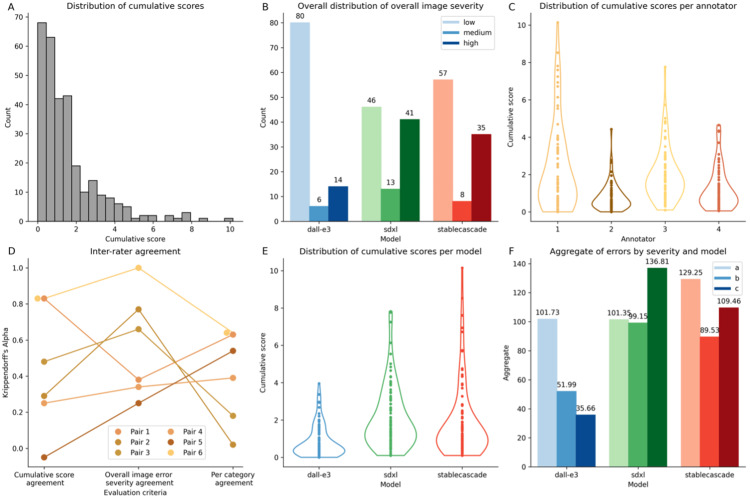
Results per annotator and model. A: Overall cumulative score distributions for annotation dataset. B: Distributions of overall image severity per model.  C: Distribution of cumulative scores per annotator. D: Pair-wise inter-rater agreement for the pairs of annotators across metrics. E: Distribution of cumulative scores per model. F: Aggregate error scores by severity per model.

The distribution of cumulative scores for all images and all annotators is shown in Figure 3A, and the distribution of overall per-annotator error severity levels by model is shown in Figure 3B. 

The distribution of scores for each of the four annotators (Figure 3C) reveals potential inter-rater differences in annotation styles which reflect the subjectivity of the task. Figure 3D shows the inter-rater agreement for individual annotator pairs for the three different metrics: cumulative score agreement, overall per-annotator error severity agreement, and agreement per category. The average Krippendorff’s alpha coefficient for the cumulative score agreement, the overall error severity agreement, and the binary category agreement are 0.44, 0.57, and 0.45, respectively. These are above random but reflect a relatively poor level of agreement, which may be expected given the complexity and subjectivity of the rating task.

Figure 3E shows the distribution of cumulative scores for images across models. DALL-E 3 has the lowest cumulative error scores overall (mean 0.82, variance 0.73; Welch t-test statistic -6.72 and -4.67; p-value 3.72E-10 and 7.12E-6; comparing DALL-E 3 to SDXL and Stable Cascade, respectively). Stable Cascade and SDXL were not significantly different (Welch t-test statistic of -1.05 and p-value 0.3), although we observed a slightly lower mean for SDXL but higher variance (mean of 2.1 and 1.8, variance of 2.72 and 3.69 for Stable Cascade and SDXL, respectively). 

We then compared the aggregate counts per error severity scores ("a," "b," and "c") across models (Figure 3F). While DALL-E 3 has a similar number of errors with severity "a" as SDXL (101.73 and 101.35, respectively), it has much fewer errors of severity "b" or "c" than the other models (severity "b": 51.99, 99.15, and 89.53 for DALL-E 3, Stable Cascade, and SDXL, respectively, severity "c": 35.66, 136.81, and 109.46 for DALL-E 3, Stable Cascade, and SDXL, respectively). Furthermore, SDXL has the highest number of severe "c" errors.

**Figure 4:**
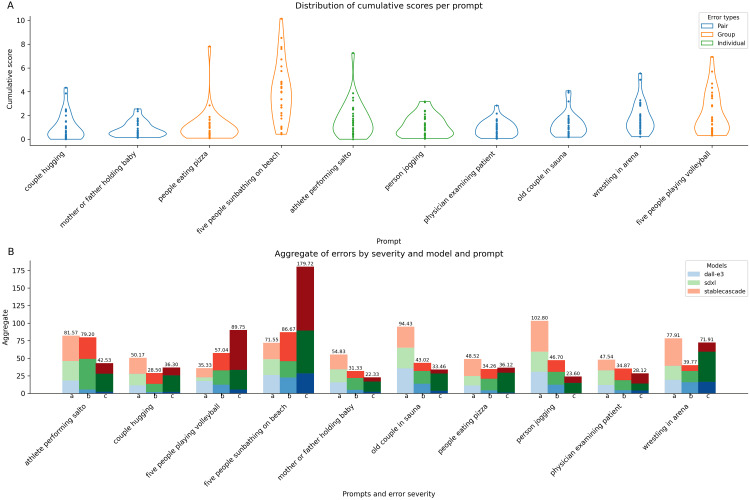
Errors per prompt. A: cumulative score, B: error aggregates per severity

Next, we compared the cumulative scores and aggregate error counts per error severity for each prompt (Figure 4). Certain prompts were more challenging and error-prone than others. For example, the prompt “five people sunbathing on a beach” led to a notably higher cumulative error score (mean 3.96, variance 6.1) than the prompt “mother or father holding a baby” (mean 0.82, variance 0.42) (Figure 4A). The difference is statistically significant (Welch t-test statistic 6.74; p-value 1.12E-7). The former prompt not only had the highest cumulative error score but also a significant number of "c" (severe) errors (Figure 4B). DALL-E 3, SDXL, and Stable Cascade exhibited numerous "a," "b," and "c" errors, with "c" errors (179.72) exceeding the cumulative counts of "a" (71.55) and "b" (86.67) errors. 

**Figure 5:**
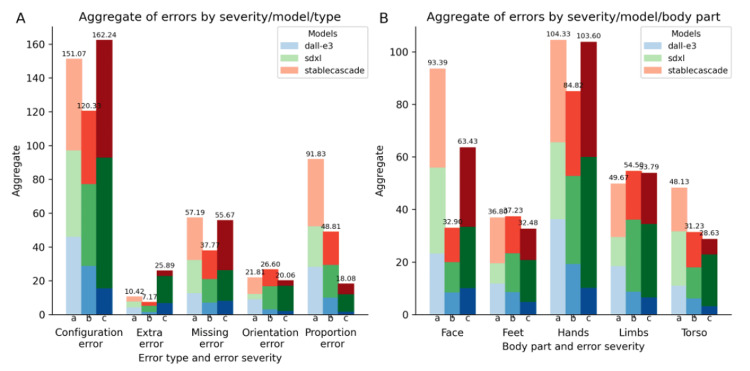
Errors by type and body part A: by error type; B: by body part

In general, prompts involving groups of people, such as “five people sunbathing on the beach” and “five people playing volleyball,” not only had higher cumulative error scores but also exhibited a more dispersed score distribution (Figure 5A). In terms of the per-severity error counts (Figure 5B), prompts featuring groups of people had more errors overall, with a higher proportion of "c" errors compared to prompts involving one or two people, although “people eating pizza” was an exception. Across all prompts, DALL-E 3 consistently demonstrated the best performance, with lower overall error counts and fewer severe "c" errors. Aside from “five people sunbathing on the beach,” DALL-E 3 showed a pattern where "a" error counts exceeded those of "b" and "c" errors. Stable Diffusion XL and Stable Cascade performed similarly; however, aside from “five people sunbathing on the beach” and “five people playing volleyball,” Stable Diffusion Cascade had lower total error counts than Stable Diffusion XL and generally fewer "c" errors. 

Next, we looked at the error distribution per error type (Figure 5).

For all models, configuration errors are the most prominent, with the count of "a," "b," and "c" levels far exceeding those of the other error types (Figure 5A). Moreover, the number of "c" errors surpasses both "a" and "b" errors, indicating that not only are configuration errors more common but also often quite severe. Missing errors follow, with similar counts of "a" and "c" errors. Surprisingly, extra errors rank last in the total count. Nonetheless, these errors are more severe when they occur. Conversely, proportion errors, despite their higher overall count, are predominantly "a" errors. For instance, many images generated by DALL-E 3 feature exaggerated muscle or body proportions without missing or extra body structures. Regarding body parts, the hands are the most problematic anatomical region (Figure 5B). The face and limbs follow, with the feet and torso being the least problematic. 

## Discussion

Generative text-to-image models have the potential for wide-ranging future applications in the medical domain, including for privacy-preserving synthetic data generation. However, it is necessary to better understand the limitations of such models and qualitatively and quantitatively assess the errors they make in generating images of human anatomy. Assessing generated content is challenging and subjective, and to the best of our knowledge, this is the first attempt to create a systematic approach to structure the evaluation of medically relevant errors with broad applicability. The most common evaluations of synthetic images relate to generated image diversity and quality with respect to, e.g., aesthetics, but not anatomical correctness. A rating of "defects" in generated images is proposed in [26], in which defects are equivalent to the errors that we include in our anatomical error classification system, but in that work, different types of defects are not further subdivided. They found a lower defect rate in MidJourney as compared to SD1.5 and SDXL. 

There are several limitations to our method. First, as tested through double annotations and quantifications of inter-rater agreement in our sample, there is a subjective aspect in how the errors are identified in the images, particularly regarding their varying severity levels. The introduction of an annotation manual, which is available from the project's GitHub repository (https://github.com/hastingslab-org/ai-human-images), aimed to reduce subjectivity by defining operative rules for how to apply the method. We also recommend training and discussions to mutually align annotators’ potentially subjective interpretations of error severities and note that a more extensive annotation training might potentially enhance inter-rater consistency beyond what we observed in our study. However, even with the manual and training, it is impossible to fully exclude subjectivity from each annotator’s decisions on what counts as an anatomical error in generated images and error severity. This is because such decisions are necessarily influenced by multiple subjective factors, such as the annotator’s implicit assumptions about what counts as "normal" anatomy or which body parts should be visible in an image from a particular perspective. Another limitation is that for images that show larger groups of multiple individuals, the application of our method’s manual error counting becomes impractical since the number of anatomical parts that must be visually examined for errors becomes too large and the body parts too small to be assessed without zooming in. For this reason, in our image dataset, we prompted a maximum of five figures (resulting in images containing up to seven figures given the variability in outputs). The more people are shown in the images, the more difficult it becomes to count the errors manually. This especially relates to "smaller" parts of anatomy, such as individual digits rather than whole hands or feet. We note that we did not count moles or other naturally occurring skin irregularities as errors, and we found no abnormal lesions on the faces in our image sample. Nevertheless, in images with more individuals, any errors in the face become more difficult to spot and assess as the faces get proportionally smaller in the image. Finally, for practical reasons, we limited the error types to five, as increasing the number of types would have led to a significant increase in error categories (number of error types x number of anatomical regions), thus potentially overwhelming the annotators. This, however, meant that some error types (e.g. configuration and proportion errors) are more broadly defined than others (e.g., missing, extra, and orientation errors).

The qualitative findings that we observed during the process of generating our dataset with the 10 prompts are fully aligned with and further contribute to the ongoing discussion in the current literature around the implicit gender and ethnic biases of generative models [27,28,29]. By prompting older people in a sauna, we have also demonstrated an implicit ageism bias in these models, as images of older individuals were often rendered with unrealistically youthful bodies, a type of bias that has to our knowledge not previously been reported in the literature. In the future, additional anatomical scenarios might be able to uncover additional limitations of the models that were not tested explicitly through our sample. For example, scenarios where people dynamically engage in some actions not tested here, such as cooking, where a lot of utensils are taken in the hands, or performing surgery, to note a more medical example. More diverse ethnic samples would also be interesting to test through explicit prompting, to observe whether they might potentially lead to different anatomical mistakes across ethnicities. It would also be interesting to test how the models perform when prompted to generate individuals with diverse anatomical pathologies, which we did not explore, as we focused on canonical anatomy for the present study.

Perhaps unsurprisingly, in our quantitative comparison, the commercial model DALL-E 3 performed better than the competing non-commercial alternatives. However, this result has to be balanced alongside qualitative observations of problems that were not covered by our anatomical error classification system, such as the ethnic bias, as well as the observed “twinning” (multiple people having the same facial features) and “hyper-idealization” (overly perfect and unrealistic representations) of human figures, which was more prominent in DALL-E.

More surprising was the result of the diachronic comparison of two versions of Stability AI’s models, the more recent Stable Cascade and the earlier Stable Diffusion XL. In our dataset, the quantitative comparison of errors showed that Stable Cascade generated a smaller amount of serious errors than Stable Diffusion XL but a comparable amount of moderate and even a larger amount of minor errors. We presume that the reason for this relative similarity of performance can be attributed to the fact that these two models use different architectures and that the Stable Cascade was primarily developed to optimize the speed, efficiency, and flexibility for fine-tuning image generation, rather than to substantially reduce errors in the generated images.

Interestingly, in developing a range of prompts that covered different everyday scenarios, we discovered that some prompts challenged all the models. For example, “five people sunbathing on a beach” led to a surprisingly high number of severe errors in all three models. Our quantitative findings also reveal that prompts that describe less common bodily constellations, such as two people wrestling in an arena or a single individual performing a salto, also challenge all the models we tested, resulting in an increase of anatomical errors compared to less challenging prompts, such as a mother or father holding a baby. This is in line with previous results evaluating generative AI models, which have shown that such models’ performance drops for prompts corresponding to scenarios less present in the training dataset [30]. 

A limitation of our findings is that our dataset was relatively small and only contained images generated by three text-to-image models using 10 prompts. In the future, a more comprehensive comparison across further models using a larger number of prompts that cover a more heterogeneous range of everyday scenarios would be desirable. However, such comparison would require a larger team of annotators, which, in turn, could potentially exacerbate the problem of limited inter-rater agreement when applying a method that necessarily entails a subjective element of image evaluation. Thus, we also plan to explore approaches that use additional model types such as segmentation to attempt to partially automate parts of the error detection and classification system.

## Conclusions

Generative AI is already having a significant impact on society and in medicine, but developing a better understanding of the strengths and limitations of such models requires new methods to evaluate their outputs. Here, we report a novel approach to evaluate errors in the images of human anatomy from such models and apply our approach to compare models and their outputs.

Our approach is as yet far from clinical applications but was developed with potential medical applications in mind in synthetic data generation and medical education, to enable systematically differentiating medically meaningful anatomical pathologies from model-driven unintended visual errors, without discriminating against persons with non-normative anatomies. The distinction of different types of errors within anatomically relevant locations and the associated scoring system has broad applicability to classify errors in other types of anatomical images. In future works, we plan to apply this method to other types of synthetic anatomical images, including retinal fundus images, which are used as a basis for diagnostic predictions. In addition, our method could support academic researchers in assessing and comparing implicit visual biases of different generative models, supporting the improvement and optimization of such models in the future.

## Appendices

Appendix 1: developing the error classification system

Model Selection

To develop a classification system tailored to evaluating technical errors in the photorealistic generative imagery of human anatomy, we started by selecting the models for our study. After investigating the different available models, we selected three different text-to-image generators: DALL-E 3, Stable Diffusion 1.5, and Stable Diffusion XL. Our choice of models aimed to include a widely used exemplar of both a proprietary (DALL-E) and an open-source (Stable Diffusion) image generation model, as well as to look at different versions of one model (Stable Diffusion 1.5 and Stable Diffusion XL) in order to take into account its continued optimization. 

Initial Image Collection

Having selected the image-to-text models, we then turned to generating a collection of images by prompting these models. First, we performed an exploratory image generation in order for the research team to familiarize themselves with the technology and to inform the prompting strategy that was used in the subsequent stages. At this point, each team member generated five photorealistic images of humans engaging in everyday situations with each of the three models and brought those images to the group discussions. Based on these discussions, we designed six simple prompts. The prompts were specifically chosen to cover different everyday scenarios that included a single individual, a couple, or a group of individuals. This choice allowed us to quantify the presence of mistakes depending on the number of individuals represented in the images. The prompts were also created to cover a variety of scenarios that resulted in visualizations of human bodies in either static (e.g., sitting) or dynamic (e.g., dancing) situations. Moreover, in addition to prompting the models to generate imagery of people in everyday environments (e.g., an apartment), we included other contexts, such as being on a beach. The latter choice allowed us to generate images revealing anatomical structures that typically remain hidden from view in other everyday scenarios. Using our six prompts, we generated 10 images per prompt with each of the three models, resulting in an initial collection of 180 images.

First Stage of Error Classification System Development

Having used the prompts to generate the image material, we then proceeded to develop our error classification system based on the comparative visual analysis of the generated images, which was performed in two stages. 

In stage 1, we introduced a preliminary typology of errors to distinguish between different kinds of unintended visual deviations from standard representations of human anatomy. Our goal was to develop categories that are mutually distinct enough so as not to overlap, while at the same time covering the entire range of anatomical errors that we identified in our image sample through visual inspection. Based on the analysis of our image collection, we defined five types of anatomical generation errors: 1) *missing errors*, designating an absent body part (e.g., an arm without a hand); 2) *extra errors*, referring to the generation of additional body parts (e.g., a hand with six fingers); 3) *configuration* *errors*, encompassing the generation of body parts that are either disjointed (e.g. a hand disconnected from the body), displaced (e.g., a hand connected to the chest instead of the arm), or fused in ways that make the differentiation of individual body parts challenging or impossible (e.g., a hand merged with a fork); 4) *orientation* *errors*, entailing anatomically implausible orientations of various body parts (e.g., the upper and lower body facing in opposite directions); and 5) *proportion* *errors*, comprising distortions arising from misshaped or disproportionate body parts (e.g., limbs that are too short for the visualized body).

In this initial stage, apart from introducing our error typology, we also topographically divided the body into five anatomical regions: 1) torso, 2) limbs, 3) feet (with five toes each), 4) hands (with five fingers each), and 5) face.

This topographic division allowed us to evaluate the presence or absence of each of our five error types in each anatomical region separately. The combined use of error types and anatomical subcategories aims to enable the assessment of how well different text-to-image models generate different body parts and potentially identify which types of mistakes more frequently occur in a given anatomical region in a particular text-to-image model.

Checking Inter-annotator Agreement

Next, we turned to evaluate the performance of this preliminary version of our error classification system with a view to optimizing it in the subsequent stage of its development. To this end, a team of five annotators applied the system to our initial collection of generative images. To test if our preliminary classification system would achieve sufficient inter-rater agreement, each of the 180 images was annotated by two annotators. We shuffled the images to ensure that each annotator received images generated by different models and stemming from different prompts. Furthermore, we devised a standardized annotation sheet in Excel. This sheet introduced a temporal sequence in which the annotation is performed both concerning the assessment of different error types and the anatomical regions. According to this predefined sequence, the annotators are required to first look for the missing and extra errors, then proceed with the configuration and orientation errors, and finish with the proportion errors. Relatedly, the annotation sheet imposes the temporal sequence in which, for each error category, the anatomical regions should be scrutinized, starting with the torso and the limbs, then moving on to the hands and the feet, and finally examining the face. In the annotation sheet, the annotator can see the image’s identification number, the corresponding prompt, and the model used to generate it. Using the annotation sheet, each annotator marked the presence or absence of a particular error type in a specified anatomical region for each of the images assigned to them.

The comparison of the annotations performed by the different annotators revealed significant disagreements in how to apply our preliminary classification system to the generated images. Based on the analysis of the annotations, we determined that the disagreement primarily stemmed from subjective decisions about the relevance of different error types and their mutual differentiations. We also established that by focusing on the mere presence or absence of a particular error type in a generative image, our preliminary system was unable to differentiate between images that contained a large number of serious errors (such as those generated by Stable Diffusion 1.5) and those that had a few minor errors (such as those generated by DALL-E 3). Thus, in the next stage, we used these findings to optimize our system.

Second Stage of the Error Classification System Development

In stage 2, we introduced a quantification of errors per image. We thus instructed the annotators to first count for each anatomical region separately how often it appears in the image and then evaluate each of the instances of that anatomical region for a particular type of error. For example, if two persons are shown in an image with their entire figures fully visible, then the annotator should separately count each error category for two trunks, four limbs, four hands and four feet, and two faces. The resulting score, expressed as a fraction, denotes how many instances of a particular anatomical region shown in the image exhibit a particular type of mistake (e.g., ¼ limbs with a proportion error). We further introduced into our method the weighting of each error through the assignment of its severity. The grading of the error severity is rated as "a" for a minor error, "b" for a moderate error, and "c" for a severe error. Each severity grade carries a different penalty score: "a" error: 0.2 points; "b" error: 0.5 points; and "c" error: 1 point. At the end of the annotations, the scores for the different error categories are multiplied by the corresponding penalty points depending on the severity grade and then added up to obtain the cumulative error score for the generated image. Thus, through this update, we obtained an evaluation method that allows a more fine-grained quantitative comparison of the quality of images generated by different text-to-image models from the perspective of the models’ limited ability to generate error-free representations of human anatomy.

Finally, we tested our updated classification method for the comparison of images generated by different text-to-image models. We aimed to establish if our classification method would allow us to quantify the level of anatomical errors across the different models based on cumulative scores. Using a proportional score ensures that we can reliably compare images that exhibit different numbers of people.

We performed another annotation round, this time focusing on a small subset of our image collection. Four annotators assessed 25 images with each image being assessed by two annotators. Our statistical analysis of the thus obtained annotation scores revealed that our optimized error classification system was able not only to quantitatively distinguish better performing generative models but also to assess which types of errors and of which severity grade were predominant in each generative model.  

## References

[1] Clusmann J, Kolbinger FR, Muti HS (2023). The future landscape of large language models in medicine. Commun Med (Lond).

[2] Chen S, Guevara M, Moningi S (2024). The effect of using a large language model to respond to patient messages. Lancet Digital Health.

[3] Topol EJ (2023). As artificial intelligence goes multimodal, medical applications multiply. Science.

[4] de Hond A, Leeuwenberg T, Bartels R (2024). From text to treatment: the crucial role of validation for generative large language models in health care. Lancet Digital Health.

[5] Zack T, Lehman E, Suzgun M (2024). Assessing the potential of GPT-4 to perpetuate racial and gender biases in health care: a model evaluation study. Lancet Digital Health.

[6] Hastings J (2024). Preventing harm from non-conscious bias in medical generative AI. Lancet Digital Health.

[7] Noel GP (2024). Evaluating AI-powered text-to-image generators for anatomical illustration: a comparative study. Anat Sci Educ.

[8] Goodfellow IJ, Pouget-Abadie J, Mirza M (2014). Generative adversarial networks. arXiv.

[9] Skandarani Y, Jodoin P-M, and Lalande A (2021). GANs for medical image synthesis: an empirical study. arXiv.

[10] Müller-Franzes G, Niehues JM, Khader F (2022). Diffusion probabilistic models beat GANs on medical images. arXiv.

[11] Fan BE, Chow M, Winkler S (2024). Artificial intelligence-generated facial images for medical education. Med Sci Educ.

[12] Koljonen V (2023). What could we make of AI in plastic surgery education. J Plast Reconstr Aesthet Surg.

[13] Kumar A, Burr P, Young TM (2024). Using AI text-to-image generation to create novel illustrations for medical education: current limitations as illustrated by hypothyroidism and Horner syndrome. JMIR Med Educ.

[14] Alberto Mazzoli C, Semeraro F, Gamberini L (2023). Enhancing cardiac arrest education: exploring the potential use of MidJourney. Resuscitation.

[15] Zhu L, Mou W, Wu K, Zhang J, Luo P (2024). Can DALL-E 3 reliably generate 12-lead ECGs and teaching illustrations?. Cureus.

[16] da Mota Santana LA, do Nascimento-Júnior EM, Floresta LG (2024). Revolutionizing oral and maxillofacial surgery: the role of DALL-E's AI-generated realistic images in enhancing surgical precision. J Stomatol Oral Maxillofac Surg.

[17] Buzzaccarini G, Degliuomini RS, Borin M (2024). The promise and pitfalls of AI-generated anatomical images: evaluating Midjourney for aesthetic surgery applications. Aesthetic Plast Surg.

[18] Cheraghlou S (2023). Evaluating dermatologic domain knowledge in DALL-E 2 and potential applications for dermatology-specific algorithms. Int J Dermatol.

[19] Pierce JE (2024). AI-generated images for speech pathology-an exploratory application to aphasia assessment and intervention materials. Am J Speech Lang Pathol.

[20] Chambon PJM, Bluethgen C, Langlotz C, Chaudhari A (2022). Adapting pretrained vision-language foundational models to medical imaging domains. arXiv.

[21] Adams LC, Busch F, Truhn D, Makowski MR, Aerts HJ, Bressem KK (2023). What does DALL-E 2 know about radiology?. J Med Internet Res.

[22] Wasielewski A (2023). Midjourney can’t count. IMAGE.

[23] Hartwig S, Engel D, Sick L (2024). Evaluating text-to-image synthesis: survey and taxonomy of image quality metrics. arXiv.

[24] Lee T, Yasunaga M, Meng C (2023). Holistic evaluation of text-to-image models. arXiv.

[25] Hayes AF, Krippendorff K (2007). Answering the call for a standard reliability measure for coding data. Commun Methods Meas.

[26] Chen M, Liu Y, Xu C (2024). Evaluating text-to-image generative models: an empirical study on human image synthesis. arXiv.

[27] Ali R, Tang OY, Connolly ID (2024). Demographic representation in 3 leading artificial intelligence text-to-image generators. JAMA Surg.

[28] Choudhry HS, Toor U, Sanchez AJ, Mian SI (2023). Perception of race and sex diversity in ophthalmology by artificial intelligence: a DALL E-2 study. Clin Ophthalmol.

[29] Offert F, Phan T (2022). A sign that spells: DALL-E 2, invisual images and the racial politics of feature space. arXiv.

[30] Ganguli D, Hernandez D, Lovitt L (2022). Predictability and surprise in large generative models. Proceedings of the 2022 ACM Conference on Fairness, Accountability, and Transparency (FAccT ’22).

